# Optimizing Paclobutrazol Application for Regulating Dwarfing in Ougan (*Citrus reticulata* cv. Suavissima): Comprehensive Insights from Growth, Photosynthesis, and Physiological Responses

**DOI:** 10.3390/plants14050763

**Published:** 2025-03-02

**Authors:** Wei Liu, Yan Tang, Zhiliang Xie, Guanghui Zeng, Tingting Wu, Jinlin Liu, Ziqi Lin

**Affiliations:** 1 Institute of Horticulture, Wenzhou Academy of Agricultural Sciences, Wenzhou 325006, China; tangyan@njfu.edu.cn (Y.T.); zengguanghui@wzvcst.edu.cn (G.Z.); 2 School of Environmental and Chemical Engineering, Shanghai University, Shanghai 200444, China; hsliuwei@shu.edu.cn; 3 College of Oceanography and Ecological Environment, Shanghai Ocean University, Shanghai 201306, China; m220501178@st.shou.edu.cn; 4 State Key Laboratory of Marine Geology, Tongji University, Shanghai 200092, China; jlliu@tongji.edu.cn; 5 Faculty of Humanities and Social Sciences, Newcastle University, Newcastle upon Tyne NE1 7RU, UK; cindylinc@outlook.com

**Keywords:** ougan, paclobutrazol, dwarfing, principal component evaluation, PLS-SEM

## Abstract

Rapid urbanization and increasing land scarcity have made urban agriculture and efficient space utilization critical directions in modern agriculture. Ougan, a fruit tree valued for both its economic and ecological benefits, holds significant promise for dwarfing cultivation techniques. In this study, a root-irrigation method was used to apply paclobutrazol at various concentrations (200, 500, 1000, 1500, and 2000 mg/L) to Ougan seedlings, with a control group for comparison. Growth parameters include an average daily increase of plant height, stem girth, new branches, and new branch girth, as well as physiological indices such as leaf SPAD values, leaf nitrogen content, net photosynthetic rate, stomatal conductance, intercellular CO_2_ concentration, and transpiration rate, were measured during both spring and summer growth periods. The results demonstrate that PBZ exerts a distinct concentration-dependent regulatory effect on Ougan growth: higher concentrations significantly inhibited plant height while promoting increases in stem diameter, with several parameters exhibiting a unimodal response. Short-term (spring) PBZ application enhanced certain photosynthetic parameters, such as net photosynthetic rate and stomatal conductance; however, prolonged exposure (summer) resulted in a decline in photosynthetic efficiency and overall leaf physiological status. Through comprehensive evaluation using principal component analysis and PLS-SEM, the 500 mg/L PBZ treatment was identified as achieving the optimal balance between growth inhibition and the maintenance of photosynthetic and nutritional status, closely approximating the ideal dwarfing effect. This study elucidates the complex regulatory effects of PBZ on the growth, photosynthesis, and carbon assimilation of Ougan through natural climate, providing robust technical parameters and theoretical support for future dwarf cultivation practices. These findings facilitate the development of dwarf fruit trees into bonsai vegetation, demonstrating significant horticultural application potential.

## 1. Introduction

Plants are fundamental to terrestrial ecosystems, serving as primary producers that convert sunlight into chemical energy through photosynthesis while also playing a crucial role in carbon sequestration and nutrient cycling [1]. The growth and development of plants are influenced by a myriad of biotic and abiotic factors, including light, water, soil nutrients, and plant hormones, which collectively dictate physiological responses [2,3]. Plant growth mainly depends on the increase in the number of cells and the increase in size, which is apparently easy to see through vision. In fact, the plant growth process is regulated by internal biological factors, especially plant growth regulators. For instance, Jasmonate (JA) biosynthesis could be rapidly induced upon wounding in plants, and JA played a central role in plant response to wounding [4]. In addition, plant regulators can also become signaling molecules, and crosstalk occurs [5]. Therefore, the addition of exogenous plant growth regulators often triggers a series of physiological feedback in plants, including feedback related to photosynthesis.

Gibberellins and auxins are key regulators of plant growth, with gibberellins promoting cell elongation and division [6]. Paclobutrazol (PBZ) is a highly effective endogenous gibberellin synthesis inhibitor. The application of plant growth regulators that inhibit gibberellin synthesis, such as paclobutrazol, can effectively slow down plant growth, control stem elongation, reduce internode length, and enhance tillering, stress resistance, and yield [7,8]. PBZ, specifically, has been shown to selectively inhibit the oxidation of kaurene to kaurenoic acid, a critical step in gibberellin biosynthesis, thereby suppressing gibberellin production [9,10]. Additionally, PBZ can reduce the synthesis or metabolism of indole-3-acetic acid, increase the endogenous abscisic acid content in plants, and modulate ethylene release, making it a commonly used growth regulator for plant dwarfing [11,12]. Recent studies have indicated that PBZ can have adverse effects on soil microbial activity and diversity [13,14]. Consequently, it is essential to strike a balance between the dwarfing effects desired for plant growth and environmental friendliness by determining the optimal concentration of PBZ. In addition, Senoo and Isoda [15] demonstrated that PBZ-driven nutrient redistribution (from vegetative to reproductive organs) significantly enhances the chlorophyll content, the quantum efficiency of photosystem II, and the CO_2_ assimilation rate of peanut. These transient improvements in photosynthetic characteristics likely provide additional carbon resources for early pod development. However, the effects of PBZ on photosynthetic physiology and its broader ecological feedback mechanisms remain poorly understood, necessitating further research.

Plant photosynthesis is an important source of plant dry matter accumulation, which is influenced by both internal regulation and external environment [16]. Net Photosynthetic Rate indicates the plant’s ability to fix carbon dioxide through photosynthesis, reflecting its growth potential and productivity, which is an important index to measure plant health and photosynthesis intensity. The key intrinsic biological regulators of photosynthesis encompass chlorophyll concentration and leaf nitrogen content. As a nitrogen-containing organic compound, chlorophyll serves as the primary pigment responsible for harvesting light energy. The porphyrin ring in its molecular structure captures light quanta to drive electron transport chains, converting solar energy into chemical energy (ATP and NADPH), making its concentration a critical determinant of photosynthetic efficiency. Furthermore, leaf nitrogen content influences photosynthesis through dual mechanisms: it provides essential nitrogen substrates for chlorophyll biosynthesis while simultaneously participating in the synthesis of photosynthetic enzymes such as Rubisco, thereby coordinately regulating carbon assimilation efficiency. The key environmental determinants of photosynthesis are atmospheric CO_2_ availability and plant–water relations. Among these, stomatal conductance quantifies the aperture of stomata, modulating bidirectional gas exchange (CO_2_ influx vs. H_2_O efflux) and serving as a critical indicator of water-use efficiency. Intercellular CO_2_ concentration reflects the CO_2_ partial pressure within the leaf mesophyll, which directly influences carboxylation efficiency by Rubisco and indirectly reveals the balance between stomatal regulation and biochemical assimilation capacity. Concurrently, the transpiration rate measures the mass flow of water vapor released through stomata, providing insights into plant hydraulic strategies and adaptive responses to drought stress.

Ougan (*Citrus reticulata* cv. Suavissima), a nutritionally and medicinally valuable citrus fruit endemic to Wenzhou, China, is rich in bioactive compounds like flavonoids and monoterpenoids, enabling applications in functional beverages and cancer-preventive therapies [17,18,19,20,21,22]. Enzymatic modification of its bitter compounds (e.g., neohesperidin conversion) further improves commercial viability in beverage industries [23]. Despite its economic promise, cultivation constraints in mountainous Wenzhou necessitate yield optimization through dwarfing germplasm development, which enhances land-use efficiency and supports sustainable urban agriculture [24]. Dwarf varieties also hold potential as ornamental potted plants, though key bottlenecks in dwarfing technology remain unresolved. Meanwhile, the effects of PBZ on plant growth, photosynthesis, and physiological processes are not clear. In this study, we aim to investigate the effects of different concentrations of PBZ on the dwarfing of Ougan, with the objectives of optimizing the cultivation mode and space utilization of Ougan. The significance of this research lies in its potential to address the knowledge gap in dwarfing Ougan cultivation mode, which is crucial for the advancement of the Ougan industry and the broader agricultural sector.

## 2. Results

### 2.1. Effect of PBZ on the Growth and Physiology of Ougan

**Figure 1:**
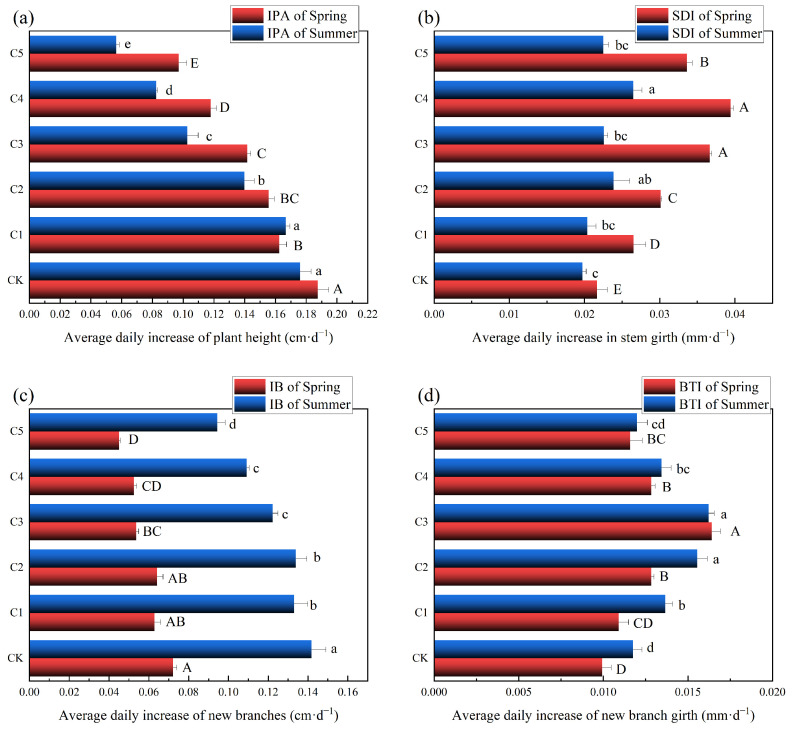
Growth indexes of Ougan under different PBZ treatments: (**a**) Plant height, (**b**) Stem girth, (**c**) new branch length, and (**d**) new branch girth. Different letters indicate significant differences; capital letters indicate differences among treatment groups in spring; lowercase letters indicate differences in summer.

As shown in Figure 1, PBZ has a significant inhibitory effect on the growth of Ougan. In spring, PBZ significantly inhibited the IPA of Ougan, and the inhibition effect was more obvious with the higher concentration, which showed a significant difference between treatment groups and control groups (*p* < 0.05). PBZ reached 1000 mg/L (C3) and above, and a significant difference in IB appeared, but the BTI reached the highest at 1000 mg/L (C3). Meanwhile, PBZ also showed a unipeak curve in stem girth, and the growth of stem girth in C4 was significantly higher than that in other groups (*p* < 0.05). The changes in summer are basically the same as in spring, but SDI increases higher in summer than in spring.

**Figure 2:**
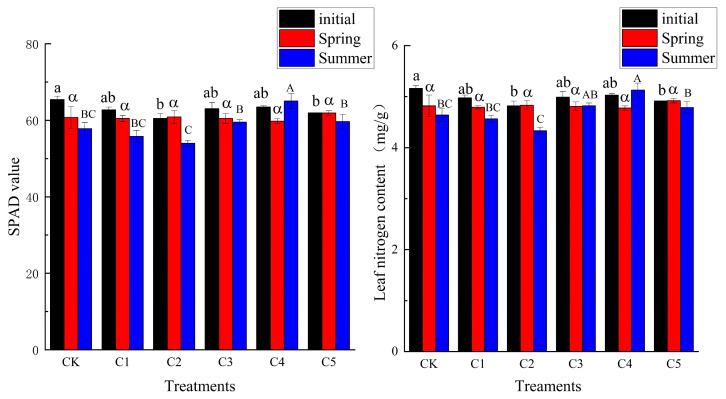
The difference in chlorophyll relative content and leaf nitrogen content under different PBZ treatments (CK: 0 mg/L, C1: 200 mg/L, C2: 500 mg/L, C3: 1000 mg/L, C4: 1500 mg/L, C5: 2000 mg/L, the same below). Different letters indicate significant differences; lowercase letters indicate differences in initial condition; Greek letter indicate differences among treatment groups in spring; capital letters indicate differences among treatment groups in summer.

As shown in Figure 2, the chlorophyll relative content (SPAD value) was basically consistent with the variation trend of leaf nitrogen content. In spring, there was no significant difference (*p* > 0.05) among the treatment groups, but there was a significant difference in summer (*p* < 0.05), with the highest in C4 and the lowest in C2.

**Figure 3:**
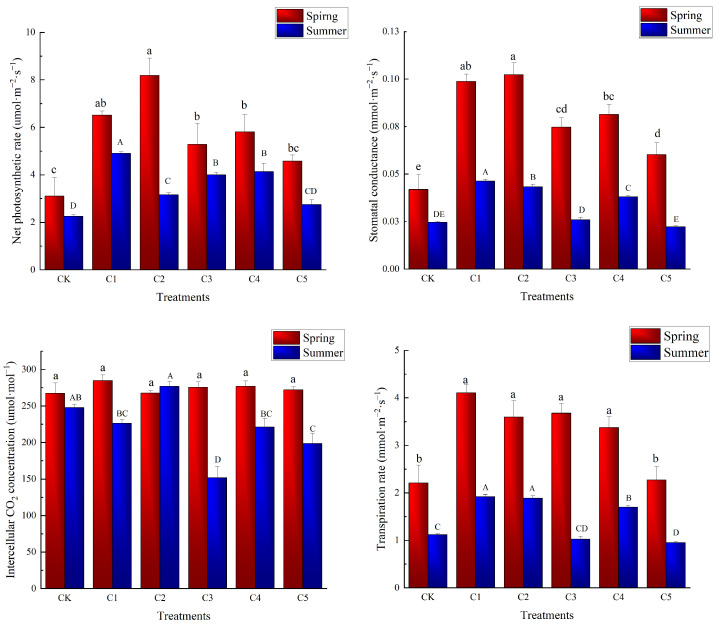
Photosynthetic physiological indexes in different treatment groups. Different letters indicate significant differences; lowercase letters indicate differences among treatment groups in spring; capital letters indicate differences in summer.

In order to better understand the effect of PBZ on the photosynthetic physiology of Ougan, we measured the relevant indexes in two seasons. The result is shown in Figure 3. In spring, there was no significant difference in intercellular carbon dioxide concentration among the treatment groups (*p* > 0.05). Still, the net photosynthetic rate, stomatal conductance, and transpiration rate of the treatment group were significantly higher than those of the control group (*p* < 0.05), except that the transpiration rate of C5 was not significantly different from that of CK. This means that the carbon assimilation ability and metabolic process of Ougan may be improved in the short term after the treatment of PBZ. The NPR levels in plants from groups C2 (8.19 umol·m^−2^ s^−1^) and C1 (6.52 umol·m^−2^ s^−1^) were statistically significantly higher than those in all other treatment groups, followed by groups C4 (5.81 umol·m^−2^ s^−1^) and C3 (5.28 umol·m^−2^ s^−1^). C5 (4.57 umol·m^−2^ s^−1^) exhibited slightly higher NPR levels than the CK (3.10 umol·m^−2^ s^−1^) but remained lower than the other treatment groups. The performance of the SC index is basically the same.

Considering that the effect on pot Ougan requires long-term observation, photosynthetic and physiological indexes of plants in summer were also measured. In summer, C2 performed poorly in terms of net photosynthetic rate, while group C1 had the highest net photosynthetic rate and stomatal conductance. C2 had the highest intercellular CO_2_ concentration, followed by CK, C1 and C4. In terms of transpiration rate, C1 and C2 show similar performance (*p* > 0.05).

### 2.2. Correlation Analysis and Principal Component Evaluation Results

**Figure 4:**
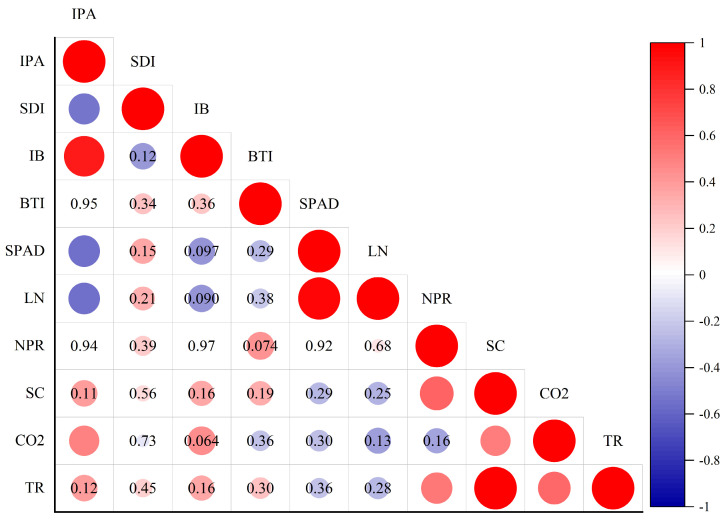
Correlation analysis for all measurement indicators. The size of the circle represents the degree of correlation, red represents the positive correlation, purple represents the negative correlation, and the significance is *p* < 0.05 for the unmarked. Numeric labels are *p*-values.

As shown in Figure 4, correlation analysis was performed for all measurement indicators. IPA and SDI showed a significant negative correlation, which indicated that PBZ inhibited the longitudinal growth of Ougan but promoted the growth of the stem girth. The correlation results showed that the collinearity of each index is low, which could be used for the principal component analysis.

The eigenvalues and variance contribution rates obtained by principal component analysis from 10 indicators are shown in Table 1. Principal components with eigenvalue greater than 1 and cumulative contribution rate greater than 80% were selected as research objectives. As shown in Table 1, the eigenvalues of the first, second, third, and fourth principal components are 4.012, 2.446, 1.458, and 1.018, respectively, and their cumulative contribution rates are 40.119%, 64.58%, 79.155 and 89.334, respectively.

The eigenvectors were calculated based on the eigenroots of the first four principal components and the load matrix (Table 2). The standardized data are marked as $u(x_{1})$ to $u(x_{10})$, respectively.

The scores of each sample in each principal component and the Synthesis scores are shown in Table 3. In this study, we expected the plant growth index to be minimum, the nutrient index, and the photosynthetic physiological index to be maximum ($u(x_{1})\cdots u(x_{4})=0,\;u(x_{5})\cdots u(x_{10})=1$), which is the ideal dwarf Ougan. The ideal score is 0.936. Therefore, the concentration of the treatment group closer to the desired score is more suitable for application in actual production to realize the evaluation of each treatment group.

As shown in Table 3, the optimal concentration of orange dwarfing effect was 500 mg/L (C2), which is the concentration closest to the ideal score. Next in importance are C1 and C4. Therefore, based on the comprehensive evaluation method of principal component analysis, the optimal concentration is C2.

The principal component matrix can also be used to measure the contribution of the principal component. Specifically, a larger absolute value of load means a larger contribution from the corresponding principal component [24]. In the first principal component, factors such as the increase of plant height, the increase of stem girth, SPAD values, leaf nitrogen content, intercellular CO_2_ concentration, and transpiration rate significantly contribute to the dwarfing of Ougan. Meanwhile, the second principal component is primarily the net photosynthetic rate influence on the dwarfing of Ougan.

### 2.3. Analysis of PBZ Effects on Plants Physiological Traits Using SEM

**Figure 5:**
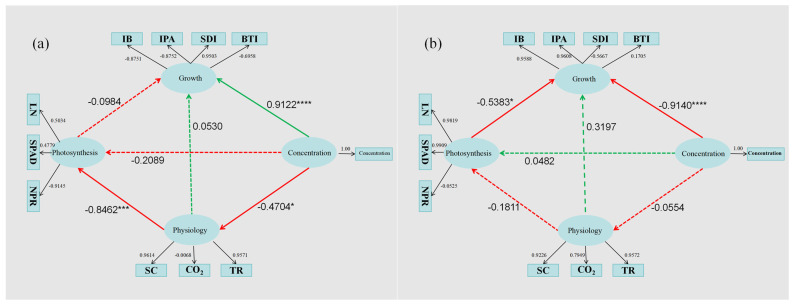
The causal relationship of effects of polybulobuzole on plants in the PLS-SEM model for (**a**) spring and (**b**) summer. The red arrow indicates a negative correlation, the green arrow indicates a positive correlation, the dotted arrow indicates that the path relationship is not significant, the solid arrow indicates that the path relationship is significant, and the significance is indicated by * (*, *p* < 0.05, ***, *p* < 0.001, ****, *p* < 0.0001).

Partial least squares structural equation modeling (PLS-SEM) is a multivariate statistical technique that helps examine complex relationships among a number of variables. PLS-SEM is a multivariate analysis technique that allows researchers to test theoretically supported causal relationships that exist among variables of interest [25]. In this study, we wanted to understand the causal relationship between the inhibition of plant growth by PBZ, so the 10 indicators measured were classified into three potential variables, namely, growth, photosynthesis, and carbon assimilation. After the index data were converted into membership function values (u(x)), the PLS-SEM model was substituted to explore the causal relationship between the factors. The result is shown in Figure 5.

In spring, PBZ significantly promoted the growth of Ougan (0.9122, *p* < 0.0001). Specifically, the growth indexes were mainly reflected in the promotion of stem girth, while the other indexes were inhibited. PBZ also significantly affected the carbon assimilation process, increasing stomatal conductance, increasing transpiration rate, and weakening carbon assimilation ability. PBZ had no significant effect on photosynthesis but promoted net photosynthesis by affecting stomatal conductance and transpiration rate, that is, the photolysis process of CO_2_ content and water in photosynthesis. However, due to the restriction of chlorophyll, PBZ still inhibited the photosynthetic process.

In summer, PBZ significantly inhibited the growth of plants (−0.9140, *p* < 0.0001), which may be the cumulative effect of PBZ’s long-term influence on the carbon assimilation process. Secondly, the photosynthetic process inhibits plant growth (−0.5383, *p* < 0.05), especially the restriction of chlorophyll and nutrient nitrogen.

## 3. Discussion

### 3.1. Influence of PBZ and Selection of Optimal Concentration

The present study aimed to investigate the effects of different concentrations of PBZ on the growth, physiology, and dwarfing of Ougan. The study establishes potted fruit trees using dwarfing techniques, creating landscape plants that make Ougan suitable as a modern city bonsai. This approach expands the original cultivation scale of Ougan, making full use of urban spaces such as the balconies of city buildings, the living rooms of citizens’ homes, and the rooftops of residential areas. Additionally, it reduces the occupation of agricultural land through potted plants, thereby addressing the issue of land use efficiency. The optimal concentration not only achieves the dwarfing effect but also ensures that the Ougan maintains strong photosynthetic capacity and good physiological condition. The findings of this study clearly demonstrate that PBZ has a significant inhibitory effect on the growth of Ougan plants. This is consistent with previous research that has shown PBZ to be an effective inhibitor of gibberellin synthesis, which in turn slows down plant growth and controls stem elongation [26]. The concentration-dependent effect of PBZ on plant height and stem girth observed in this study suggests that higher concentrations of PBZ lead to more pronounced growth inhibition. The unimodal response curve observed for stem girth in relation to PBZ concentration indicates an optimal point at which the growth response is maximized. This could be attributed to the complex interplay between gibberellins and other plant hormones, such as auxins, which are known to be influenced by PBZ [27]. The peak effect at 1000 mg/L (C3) and the subsequent decline at higher concentrations may reflect a threshold beyond which the inhibitory effects of PBZ on gibberellin synthesis outweigh any potential growth-promoting effects. It is worth mentioning that in the spring and summer of this study, the net photosynthetic rate of all treatment groups was significantly higher than that of CK, reflecting that PBZ can significantly improve plant photosynthetic capacity, which is consistent with previous studies [28,29]. It is important to note that while C4 and C5 show significant dwarfing effects, they also result in a decrease in the plant’s net photosynthetic rate, which could adversely affect fruit set and sweetness. Therefore, the selection of the optimal PBZ concentration must consider not only the dwarfing effect but also the potential impact on fruit quality and yield. The optimal concentration of PBZ for Ougan dwarfing, as determined by the synthesis score, is 500 mg/L (C2). This concentration is closest to the ideal score, indicating that it provides the best balance between growth inhibition and maintenance of photosynthetic performance. The strong heterogeneity among the data indicates that multiple factors are influencing plant response to PBZ, and these factors are not uniformly correlated, highlighting the complexity of plant growth regulation.

### 3.2. Impact of PBZ on Physiology and Leaf Nitrogen Content

The SPAD values, which measure the relative content of chlorophyll, show a consistent trend with the nitrogen content in leaves, likely because nitrogen is required to form the porphyrin ring structure during the biosynthesis of chlorophyll, which is the core part of the chlorophyll molecule [30]. SPAD and leaf nitrogen content are crucial for understanding the impact of PBZ on photosynthetic capacity. The lack of significant difference among treatment groups in spring suggests that PBZ may not immediately affect chlorophyll synthesis [31]. However, the significant difference observed in summer indicates a long-term impact of PBZ on chlorophyll content, which could be related to the overall health and photosynthetic efficiency of the plants [32]. The highest chlorophyll content in C4 and the lowest in C2 suggest that intermediate concentrations of PBZ may be more conducive to maintaining photosynthetic pigment levels [33]. Leaf nitrogen content is a critical indicator of plant nutritional status and plays a significant role in photosynthesis and overall plant growth. The significant difference in leaf nitrogen content among treatment groups in summer implies that PBZ affects nitrogen uptake or utilization, which could have implications for fruit quality and yield [34]. The interaction between PBZ and nitrogen metabolism warrants further investigation, as it could provide insights into the nutritional management of Ougan trees under PBZ treatment. The net photosynthetic rate, stomatal conductance, and transpiration rate are key parameters in understanding the photosynthetic physiology of plants. The enhancement of these parameters in the presence of PBZ, particularly at lower concentrations (C1 and C2), suggests a short-term positive effect on carbon assimilation and metabolic processes. This could be due to the increased availability of resources for photosynthesis, as PBZ is known to reduce plant size and, thus, potentially concentrate nutrients in the remaining biomass [20]. However, the long-term effects of PBZ, as observed in summer, indicate a decline in photosynthetic performance, particularly in C2, which had the highest intercellular CO_2_ concentration. This suggests that while PBZ may initially stimulate photosynthesis, it could eventually lead to a downregulation of photosynthetic capacity, possibly due to resource limitations or alterations in stomatal regulation.

The PLS-SEM analysis provides a causal framework for understanding the complex relationships between PBZ treatment and plant growth, photosynthesis, and carbon assimilation. The significant promotion of growth by PBZ in spring, particularly in stem girth, suggests that PBZ may initially stimulate certain growth processes. However, the long-term inhibitory effect observed in summer indicates that PBZ’s impact on carbon assimilation and photosynthesis may eventually override any initial growth promotion, leading to overall growth inhibition. The causal relationships revealed by the PLS-SEM model highlight the importance of considering the timing and duration of PBZ application. The initial stimulation of growth and photosynthesis may be beneficial for resource allocation and early season growth, but prolonged exposure to PBZ could lead to negative effects on photosynthesis and carbon assimilation, which are crucial for fruit development and quality.

## 4. Materials and Methods

### 4.1. Plant Material and Experiment Design

In this study, the Ougan plant seedlings are produced through plant grafting, are 2 years old, and are provided by the Wenzhou Academy of Agricultural Sciences (Wenzhou, China). In November 2022, the seedlings were placed in a shaded area for two weeks to allow acclimatization. Ninety-gallon pots (with an outer diameter of 28 cm, inner diameter of 26 cm, and height of 30 cm) were prepared. A mixture was made of perlite, peat, and yellow mud in a ratio of 1:2:4 to serve as the substrate soil for cultivating the seedlings. After incorporating 0.5 L (concentration is 6.67 g/L) potassium fulvic acid per pot (as fertilizer), the mixture was filled into the gallon pots to the same height (25 cm).

The experiment was conducted outside the greenhouse of the Wenzhou Academy of Agricultural Sciences on 11 April 2023. Each gallon pot was planted with one Ougan plant. At the start of the experiment, 200 mL of PBZ was added at different concentrations via root irrigation, specifically 200 mg/L, 500 mg/L, 1000 mg/L, 1500 mg/L, and 2000 mg/L, totaling five concentration gradients, which were sequentially labeled as C1, C2, C3, C4, C5. An equal amount of pure water was poured into the control group, labeled as CK. Each treatment group was set up with 18 plants (*n* = 18), and PBZ was applied only once. Following the transfer of plants from the greenhouse to experimental conditions, daily meteorological parameters were recorded throughout the experimental period, including maximum air temperature (°C), minimum air temperature (°C), mean relative humidity (%), and average solar radiation (W/m^2^/day). The complete dataset is available in Supplementary Materials.

### 4.2. Measurement Indicators and Methods

On the day prior to the application of PBZ, the plant height, stem girth of trunk, and the length and thickness of new branches of the Ougan plants were measured using a tape measure and a caliper. Chlorophyll relative content and leaf nitrogen content were determined from randomly selected mature leaves in the middle section using Chlorophyll Meter SPAD-502 and a plant nutrition tester (TYS-3N, Tuopu, Hangzhou, China). The measured indicators serve as the initial data.

Spring is the period of vigorous growth for Ougan seedlings. Forty days after the application of PBZ, the Ougan plants were measured for the aforementioned indicators (22 May). Summer marks the vegetative growth peak period for Ougan, at which time (30 August, 100 days after treatment) the indicators were measured again to analyze the impact of PBZ on plant growth and photosynthetic pigments. Each index measured included an average daily increase of plant height (IPA), average daily increase of stem girth (SDI), average daily increase of new branches (IB), average daily increase of new branch girth (BTI), SPAD value, and leaf nitrogen content (LN).

In order to explore the impact of PBZ on plant photosynthetic and respiratory processes, we also utilized the Li-6800 portable photosynthesis system (LI-COR, USA) to measure the net photosynthetic rate (NPR), stomatal conductance (SC), intercellular CO_2_ concentration (CO_2_), and transpiration rate of the plants (TR) in spring and summer. When measuring photosynthetic indicators, it is necessary to conduct the measurements after dawn but before the sun rises, as each plant leaf needs to undergo dark adaptation. Therefore, three plants were randomly selected in each group, marked on the leaves, and the same leaves were measured each time. The photosynthetic parameters were set as follows: the CO_2_ concentration in the leaf chamber was maintained at 400 μmol/mol, with illumination provided by a built-in red-blue light source at a photosynthetic photon flux density (PPFD) of 800 μmol/m^2^·s, and the leaf chamber temperature was stabilized at 25 °C.

### 4.3. Data Processing and Statistical Analysis

During the research process, Microsoft Excel 2021 (Microsoft Corporation, USA) was used for data organization, SPSS 26.0 (IBM, USA) statistical software was employed for one-way ANOVA and principal component analysis, Origin 2022 (OriginLab, USA) was utilized for creating bar charts, and Partial least squares structural equation model (PLS-SEM) was accomplished using the R version 4.3.1 (R Foundation for Statistical Computing, Austria).

The calculation formula for the membership function is as follows: where $x_{i}$ represents the measurement value of the i indicator for each treatment; $x_{\min}$ and $x_{\max}$ are the minimum and maximum values of the i indicator, respectively; $u(x_{i})$ is the calculated membership function value for the indicator.

The general form of a linear membership function can be expressed as follows:

(1) $$u\left(x_{i}\right)=\frac{x_{i}-x_{\min}}{x_{\max}-x_{\min}}$$

This formula calculates the degree of membership within a range from 0 to 1, where 0 indicates the least membership (below the minimum value), and 1 indicates full membership (at or above the maximum value). This is useful in fuzzy logic to quantify the degree to which an element belongs to a particular set.

After correlation analysis on all measured variables using SPSS, principal component analysis, and comprehensive evaluation is carried out, the proportion of the eigenvalue corresponding to each principal component to the sum of the eigenvalues of the extracted principal components is used as the weight.

(2) $$\lambda j=Bj/\sum_{j=1}^{m}Bj,\;j=1,2,3,\cdots m$$

(3) $$kj_{i}=Aj_{i}/\sqrt{Bj},\;j=1,2,3\cdots m,\;i=1,2,3\cdots n$$

(4) $$Fj=\sum_{i=1}^{n}\left(kj_{i}\times ux_{i}\right),\;j=1,2,3\cdots n$$

(5) $$F=\sum_{j=1}^{m}\left(Fj\times \lambda j\right),\;j=1,2,3\cdots m$$

*λj* represents the principal component weight, *Bj* represents the eigenvalue of the *j* principal component, *kj_i_* represents the eigenvectors value of factor i from the *j* principal component, *Aj_i_* represents loads of factor i from principal component *j*, *Fj* represents a score of factor i, uxi represents the value of calculation formula, *F* is the combined score of all principal components.

Given the multivariate evaluation framework of this study, we established a desired scoring criterion. This criterion is mathematically defined as the theoretically optimal composite score derived from PCA, representing Ougan plant samples that simultaneously achieve the minimum IPA, IB, SDI, BTI, and maximum SPAD value, LN, NPR, SC, CO_2,_ and TR.

## 5. Conclusions

This study has conducted an in-depth investigation into the effects of PBZ on the growth, photosynthesis, and physiological indicators of Ougan, revealing the interrelationships between the photosynthetic, physiological, and growth processes. Therefore, the principal component evaluation method was employed to determine the optimal concentration of PBZ for dwarfing Ougan, which is 500 mg/L. This approach not only pursues the best dwarfing effect but also takes into account the photosynthetic and physiological activities of Ougan.

## Figures and Tables

**Table 1:** The total variance is explained by each component.

Component	Initial Eigenvalue			Extraction Sums of Squared Loads		
	Total	Variance Contribution Rate	Cumulative Variance Contribution Rate (%)	Total	Variance Contribution Rate	Cumulative Variance Contribution Rate (%)
1	4.012	40.119	40.119	4.012	40.119	40.119
2	2.446	24.461	64.58	2.446	24.461	64.58
3	1.458	14.575	79.155	1.458	14.575	79.155
4	1.018	10.179	89.334	1.018	10.179	89.334
5	0.698	6.981	96.316			
6	0.315	3.152	99.467			
7	0.029	0.291	99.758			
8	0.018	0.179	99.937			
9	0.004	0.044	99.981			
10	0.002	0.019	100			
Extraction method: principal component analysis (PCA).						

**Table 2:** Principal component load matrix and eigenvectors of all measurements.

Variables	Component							
	1		2		3		4	
	Loads	Eigenvectors	Loads	Eigenvectors	Loads	Eigenvectors	Loads	Eigenvectors
IPA	0.839	0.419	−0.32	−0.205	0.069	0.057	0.357	0.353
SDI	−0.31	−0.155	0.664	0.425	0.125	0.104	−0.447	−0.443
IB	0.765	0.382	−0.219	−0.140	0.02	0.017	0.408	0.404
BTI	0.264	0.132	0.496	0.317	−0.637	−0.528	−0.033	−0.033
SPAD	−0.727	−0.363	0.287	0.184	0.474	0.393	0.347	0.344
LN	−0.748	−0.373	0.303	0.194	0.37	0.306	0.424	0.420
NPR	0.189	0.094	0.803	0.513	−0.269	−0.223	0.36	0.357
SC	0.722	0.36	0.634	0.405	0.213	0.176	−0.01	−0.01
CO_2_	0.633	0.316	−0.151	−0.097	0.671	0.556	−0.297	−0.294
TR	0.714	0.356	0.605	0.387	0.319	0.264	−0.064	−0.063

**Table 3:** The final score of each sample and the evaluation of the optimal concentration.

Sample	Y1	Y2	Y3	Y4	Synthesis Score	Mean Score and Rank of Each Groups
C1-1	1.0521	1.4578	0.8893	0.5437	1.079	0.996 (II)
C1-2	0.8455	1.2068	0.7482	0.3601	0.873	
C1-3	1.2690	1.1946	0.4130	0.6171	1.035	
C2-1	1.2339	0.8897	0.6221	0.2264	0.925	0.958 (I)
C2-2	1.2448	0.9941	0.8349	0.1419	0.984	
C2-3	1.3071	1.0636	0.7148	−0.2459	0.967	
C3-1	0.4000	1.0274	0.1281	0.6663	0.558	0.450 (V)
C3-2	0.2578	0.9943	−0.0365	0.7666	0.469	
C3-3	0.0922	0.9890	−0.2818	0.4942	0.323	
C4-1	0.3770	1.5712	0.7741	0.7366	0.810	0.766 (III)
C4-2	0.5717	1.6304	0.6439	0.3429	0.847	
C4-3	0.0570	1.3173	1.1920	0.5163	0.640	
C5-1	−0.1151	0.4778	0.3878	0.1446	0.159	0.193 (VI)
C5-2	−0.2156	0.6589	0.6246	0.1949	0.208	
C5-3	−0.0272	0.6909	0.1666	0.0698	0.212	
CK-1	0.7810	0.4628	0.7527	0.4074	0.647	0.567 (IV)
CK-2	0.7977	0.2172	0.4759	0.2454	0.523	
CK-3	0.6647	0.1507	0.6741	0.7074	0.530	
**Desired score**	0.3909	1.5863	1.4725	0.7533	0.936	0.936
Weight	0.4491	0.2738	0.1632	0.1139	-	

## Data Availability

The raw data supporting the conclusions of this article will be made available by the authors upon request.

## Supplementary Materials

The following supporting information can be downloaded at: https://www.mdpi.com/article/10.3390/plants14050763/s1, Table: Climate information during the experiment.

## Abbreviation

IPA	Average Daily Increase of Plant Height
SDI	Average Daily Increase of Stem Girth
IB	Average Daily Increase of New Branches
BTI	Average Daily Increase of New Branch Girth
SPAD	SPAD Value (An Index Used to Measure The Relative Content of Chlorophyll in Plant Leaves)
LN	Leaf Nitrogen Content
NPR	The Net Photosynthetic Rate
SC	Stomatal Conductance
CO_2_	Intercellular CO_2_ Concentration
TR	Transpiration Rate of The Plants
